# The impact of instructional behaviors on learning motivation via subjective task value in high school students in Cambodia

**DOI:** 10.1038/s41598-025-02147-z

**Published:** 2025-05-19

**Authors:** Daro Ruos, Sereyrath Em, Phanommas Bamrungsin, Buratin Khampirat

**Affiliations:** 1 Kandal Regional Teacher Training Center (RTTC), Kandal, Cambodia; 2 Institute of Social Technology, Suranaree University of Technology, Nakhon Ratchasima, 30000 Thailand

**Keywords:** Learning motivation, Subjective task value, Intrinsic motivation, Extrinsic motivation, Instructional behaviors, Structural equation modeling, Psychology, Environmental social sciences

## Abstract

Instructional behavior plays a key role in learning motivation. In many developing countries, students’ learning motivation needs to be restored, primarily through effective teaching. This research investigated the impact of instructional behaviors on learning motivation among high school students in Cambodia, emphasizing the mediating role of subjective task value. This study was conducted in three provinces of Cambodia, and a sample was obtained by convenience sampling. A total of 515 participants (42.72% male and 56.70% female) were lower secondary and high school students. Structural equation modeling (SEM) was applied to test the proposed relationship between the two first-order constructs of instructional behaviors and learning motivation. The results revealed direct positive associations between all of the constructs in the model. Autonomy support was a significant predictor of both subjective task value and intrinsic motivation. Cooperative learning support was significantly associated with only subjective task value and had no direct effect on intrinsic motivation. Conversely, video lecture support positively predicted extrinsic motivation. Additionally, subjective task value played an important role as a mediator of the relationship between autonomy support and cooperative learning support. These results emphasize the importance of developing instructional behaviors to support students’ learning motivation.

## Introduction

Flexible education to cope with difficult and restrictive situations is the most important thing to implement instantly^1,2^. Flexible education is able to rapidly help face challenging times and tasks to restore the educational system, which increases the human capital linked to social needs and global trends^3,4^. The need- and trend-related education of society plays a crucial role in achieving the economic goals of each country^5^. Developing countries are hindered by various obstacles more than developed countries are during difficult times^6,7^. Hence, flexible education should be supervised simultaneously but this is difficult in developing countries^8^. The gap between developed and developing countries might be much narrower if all stakeholders jointly concentrate and show resolve, especially concerning teacher instructional methods. Specifically, students’ educational outcomes are greatly affected by teaching behavior through efficient instructional methods^9^.

During and after the COVID-19 pandemic, many countries faced a lack of ample support for academic study^10^. Students experienced a decline in academic motivation, potentially due to the rapid changes in learning contexts and formats brought by the pandemic^11^. Students with low learning motivation and those from low-income families experienced the most hardships during the COVID-19 pandemic^12^. Students may lack the motivation to succeed in their studies if they do not perceive the value of learning or if teachers fail to create an engaging and supportive learning environment^13^. Students will be pessimistic after learning without motivation. Motivation plays a crucial role in effective learning, as it not only enhances academic performance but also promotes positive behavior and a fulfilling student experience. Understanding how to inspire children and young people to learn is essential for providing them with the best foundation for success in life^13^. Conesa et al.^14^ conducted an analysis of the basic psychological needs of students in classrooms at the elementary and secondary levels. The findings highlight the crucial role of teachers in addressing and supporting students’ psychological needs to foster an effective learning environment. Many studies suggest that to promote learning motivation in various situations, including difficult times, flexible instructional behaviors may be appropriate^1,11^. Thus, teachers’ teaching behavior is an important predictor of students’ academic motivation and positive learning outcomes. Therefore, teachers should adopt effective teaching strategies, such as autonomy support^15,16^, video lecture support^17^, and cooperative learning^18^ to increase learners’ motivation, particularly in challenging educational contexts such as the recent COVID-19 pandemic and in online courses^19,20^.

Enhancing learning motivation among high school students requires effective teaching strategies that promote spontaneous engagement and intrinsic interest. According to existing studies, one of the most effective teaching strategies is based on self-determination theory (SDT)^21^. To promote students’ intrinsic motivation, SDT highlights the importance of meeting their core psychological demands for autonomy, competence, and relatedness. Students’ willingness to study can be significantly increased by teachers who offer education that encourages autonomy, cultivates a sense of cooperative learning, provides technologies for learning, and provides an environment that encourages students to use such technologies^22^. These strategies work especially well for assisting high school students in managing their learning aspirations and encouraging sustained academic achievement. Learners who become accustomed to support through that instructional behavior have higher intrinsic and extrinsic motivations.

Additionally, some studies have explored the role of instructional behaviors and digital technologies in enhancing students’ learning motivation and academic outcomes^23^. These studies focus primarily on the direct impacts of instructional strategies and technological tools on motivation. However, few studies have examined a comprehensive framework incorporating multiple instructional dimensions or investigating how instructional behaviors influence subjective task value and intrinsic and extrinsic motivations. This is particularly the case with studies on subjective task value as a mediating variable. To address these gaps, this study conceptualizes learning motivation by examining its relationship with instructional behaviors, specifically autonomy support, video lecture support, and cooperative learning. The aim is to understand how these instructional behaviors contribute to shaping subjective task value and intrinsic and extrinsic motivations, which are key components of learning motivation.

## Rationale and research objectives

Restoring students’ learning motivation after the COVID-19 pandemic should be considered a key focus in designing effective instructional strategies to prepare for future crises. Previous studies have identified autonomy support as one of the strongest predictors of motivation^15,16^, although its influence may vary across different contexts, as shown in prior research. Additionally, video lecture support^24^ and cooperative learning methods^25,26^ have been linked to enhanced learning motivation. In light of these findings, this research seeks to address the following practical and effective research objectives.

### Objectives

This study investigates the relationship between efficient instructional behavior and learning motivation among school students in Cambodia. Specifically, it examines whether subjective task value mediates the effects of different dimensions of instructional behavior on learning motivation.

## Literature review

### Learning motivation

Motivation theories break down motivation into intrinsic and extrinsic motivations and subjective task value. According to SDT^27^, intrinsic motivation refers to whatever inspires people to learn or work internally, such as curiosity, self-competence, self-interest, and mastery learning. In contrast, extrinsic motivation refers to whatever inspires people to learn or work externally, such as punishment, pressure, grades, self-expression, and other forms of persuasion^27,28^. When learners perceive intrinsic motivation, they consistently endeavor to perform tasks and engage in learning procedures^29^. Intrinsic motivation encourages learners to engage in learning with enthusiasm, positive emotions, and self-growth, primarily when supported by a growth mindset. It fosters more profound learning and self-regulation^21,28^. However, extrinsic motivation, such as rewards or recognition, is essential to initiate engagement at the early stages- particularly for those without prior academic success. These external motivators can eventually be internalized, leading to more autonomous learning. Therefore, effective education should balance both intrinsic and extrinsic motivation to support learners throughout their development^21^.

Grounded in expectancy-value theory, subjective task value refers to a type of internal motivation driven by the degree of utility, attainment, and intrinsic value^30^. When learners perceive academic education, including task value, they gain pleasure and usefulness and set an explicit goal for their future^30^. Chan et al.^31^ argued that learning motivation grounded in SDT is associated with subjective task value grounding expectancy-value theory. Theorists also argue that when intrinsic value forms, intrinsic motivation is grown, whereas when attainment and utility value form, extrinsic motivation is grown^32^. Several previous studies revealed that intrinsic and extrinsic motivations are associated with subjective task value^33,34^. EVT suggests that motivation depends on students’ expectations of success and the value they place on tasks, including attainment, utility, intrinsic value, and cost^30^. In contrast, SDT emphasizes fulfilling psychological needs—autonomy, competence, and relatedness—as the basis of motivation^21^. SDT is a precursor to EVT, as supportive environments that satisfy these needs help students build self-determined motivation, enhancing their task value and success expectations. For instance, when teachers support autonomy and competence, students are more likely to believe in their abilities and value their learning, linking both theories in a developmental sequence of motivational growth^30,32^.

### Instructional behaviors

Instructional behavior improves learners’ proficiency through effective teaching methods^23^. Centered on previous studies and original theory, efficient instructional behaviors are divided into three categories: autonomy support, learning structure, and involvement^35^.

In this study, autonomy support refers to the extent to which teachers encourage students to take ownership of their learning by providing choices, fostering independence, and supporting self-directed decision-making^36^. This conceptualization is rooted in SDT^21^, which defines autonomy as a core psychological need essential for intrinsic motivation. However, within the framework of instructional behaviors, autonomy support specifically refers to teacher-driven strategies that promote student agency in learning environments, such as allowing students to select assignments, choose group members, or express opinions about classroom activities^37,38^. Instructional autonomy support focuses on the external role of teachers in facilitating student autonomy within structured educational settings^39^.

Learning structure refers to how well teachers convey learning information through explicit instruction by encouraging them to use follow-up learning strategies^40^. For example, learners might be motivated when offered explicit support through mechanical systems in the teaching methodology^41^. Video lecture support is an influential learning structure that promotes student learning and engagement, and might be important teaching material for increasing classroom progress and for having fascinating teaching techniques^42^. Videos linking clear textual and visual support tools encourage students to be motivated to learn and to be engaged through entire online and offline class activities^43,44^. Despite growing interest in using videos to support instruction in learning, many schools in developing countries and vocational education sectors still have little understanding of how different online video types or styles can facilitate student learning^45^.

Learning involvement refers to the degree of self-connection between learners, as well as between learners and teachers collaboratively in the learning process^46^. Previous studies have considered cooperative learning as a type of learning involvement^47^. Teachers’ consistent involvement improve students’ social skills^48^. Previous studies have revealed that cooperative learning is the most important predictor of intrinsic motivation and subjective task value^47^.

### Conceptual framework

**Fig. 1:**
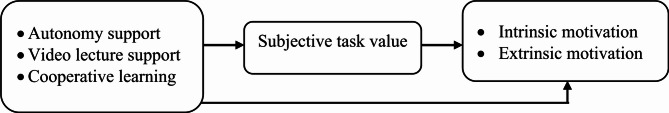
Conceptual framework of the relationship between the first-order constructs instructional behaviors and learning motivations.

On the basis of the theoretical foundation and previous research discussed above, this study formulated the conceptual framework shown in Fig. 1. This research seeks to fill gaps in existing research by investigating the effects of first-order constructs of instructional behaviors (autonomy support, video lecture support, and cooperative learning) on first-order constructs of learning motivations (subjective task value, intrinsic motivation, and extrinsic motivation). Additionally, this study examines the moderating role of subjective task value in these relationships.

In the hypothetical model, this study initially assumed that effective instructional behaviors directly influence motivation based on established theories and empirical evidence in educational psychology. Research in STD^28,49^ and EVT^32^ suggests that instructional behaviors directly impact students’ motivation. Studies have consistently demonstrated that teacher behaviors that promote a positive learning environment, support student autonomy, and reinforce task value lead to increased motivation and engagement^50^.

## Methods

### Participants

This study employed a quantitative cross-sectional research design. Convenience random sampling was used to select lower secondary and high school students from three provinces in Cambodia. For this analysis, the participants consisted of a total of 515 students; 42.72% were male, and 56.70% were female. They had been studying in grades 8 (*n* = 166, 32.23%), 9 (*n* = 138, 26.80%), and 10 (*n* = 211, 40.97%). The average age of the participants was 15.08 years (*SD* = 1.08), ranging from 13 to 18 years. The sample size was determined by the rule of thumb to select an appropriate sample size. According to previous research^51^, a suitable sample should consist of five to ten times the number of items in the research model.

### Data collection

In support of this study, data were gathered through a survey of high school students in Cambodia by using a self-report questionnaire and were collected between 1 February and 28 February 2023. Ethical approval for this study was obtained from the research ethics committee of the Royal University of Phnom Penh, Cambodia (136/2023 RUPPKS). A permission letter was sent to the principals of the high schools to request collaboration. Students were free to withdraw their participation in the questionnaire at any time. The data collected from the volunteer students were kept confidential, and only the research team had access to the data. The privacy and anonymity of the participants were maintained throughout the study period.

### Instruments

#### Learning motivation scale

To assess the students’ perceptions of learning motivation, the learning motivation questionnaire of Pintrich et al.^52^ was adapted for this study. Pintrich’s Motivated Strategies for Learning Questionnaire (MSLQ) is designed to assess different aspects of student motivation and aligns closely with SDT, especially in how it distinguishes between types of motivation. While SDT highlights the role of intrinsic motivation, which stems from fulfilling basic psychological needs, the MSLQ captures both intrinsic and extrinsic motivation. This makes it a valuable tool for understanding students’ motivational experiences, often shaped by teaching practices and the learning environment.

This instrument consists of 16 items with three subscales (Table 1), namely, (a) subjective task value (six items, e.g., “I think the course material is useful for me to learn”), (b) intrinsic motivation (five items, e.g., “I prefer course material that arouses my curiosity, even if it is difficult to learn”), and (c) extrinsic motivation (five items, e.g., “The most important thing for me is improving my overall grade or grade point average”). The item scores were averaged to create a score for each aspect for each respondent. Higher scores indicated higher learning motivation. The Cronbach’s alpha coefficients for subjective task value (0.73), intrinsic learning motivation (0.63), and extrinsic learning motivation (0.81) indicated good internal consistency (Table 2).

**Table 1:** Constructs, dimensions, and items used in this study.

Constructs	Dimensions	Items
Instructional Behavior Scale		
	Autonomy support (Autonomy)	AS1 My teacher decided with me on who I learn or do group work with.
		AS2 My teacher let me choose homework or exercise that matches my own interests.
		AS3 My teacher accepted my suggestions on how to do homework or exercises that I sought.
		AS4 My teacher accepted my suggestions on whom I prefer to do group work with.
	Video lecture support (Video)	VL1 I like viewing preclass videos better than reading text materials.
		VL2 The videos were helpful because I could do them on my own time.
		VL3 I think video-lecture learning was easy.
		VL4 The lessons were well explained in the video lecture.
		VL5 The video lectures were helpful for completing homework or exercises.
		VL6 The videos lectures were more helpful for completing the in-class activities.
	Cooperative learning support (Coop)	SC1 When I did group work, I discussed my ideas with other students in my group.
		SC2 When I did group work, I tried to understand other students’ ideas in my group.
		SC3 When I did group work, I taught or helped other students in my group when they encountered problems with some point in groupwork.
		SC4 When I did group work, I got constructive feedback from other students in my group.
		SC5 When I did group work, I collaborated with other students in my group to prepare group work.
Learning Motivation Scale		
	Subjective task value (Task)	SV1 I think I will be able to use what I learn in my school to achieve in the future.
		SV2 I think it is important for me to learn the course material for my future.
		SV3 I am very interested in each course material.
		SV4 I think the course material is useful for me to learn.
		SV5 I like learning the course material in my school.
		SV6 I think that understanding the course material is very important to me.
	Intrinsic motivation (INMO)	IM1 I prefer course material in which I can learn a new thing.
		IM2 I prefer course material that arouses my curiosity, even if it is difficult to learn.
		IM3 I am mostly satisfied that I am learning the course material as much as I can.
		IM4 I choose homework or exercises that I can do, even if I’m not guaranteed to get a good grade.
		IM5 I choose the course material that I can learn from, even if I’m not guaranteed to understand it.
	Extrinsic motivation (EXMO)	EM1 The most satisfying thing for me is getting a good grade.
		EM2 I want to get a better grade than most of the other students in the class.
		EM3 I want a perfect grade to show my ability to others in this class.
		EM4 The most important thing for me is improving my overall grade or grade point average.
		EM5 My main concern is getting a perfect grade in this class.

**Table 2:** Descriptive statistics, *ω*,* α*, AVE and CR.

Constructs	M	SD	SK	KU	α	ω
Autonomy support	3.471	0.664	-0.307	0.290	0.72	0.68
Video lecture support	3.434	0.615	-0.375	1.094	0.79	0.78
Cooperative learning support	3.948	0.465	-0.171	0.831	0.76	0.77
Subjective task value	4.151	0.406	0.263	-0.384	0.73	0.73
Intrinsic motivation	3.934	0.437	-0.055	0.359	0.63	0.62
Extrinsic motivation	3.773	0.630	-0.523	1.027	0.81	0.81

*M* = mean; *SD* = standard deviation; *SK* = skewness; *KU* = kurtosis; *α* = Cronbach’s alpha coefficient; *ω* = McDonald’s omega coefficient; AVE = average variance extracted; CR = composite reliability.

#### Instructional behavior scale

The instructional behaviors scale was adapted from prior studies^31,47,53^. This measurement consists of 15 items that measure 3 components (Table 1), including (a) teachers’ autonomy support (four items, e.g., “My teacher accepted my suggestions on how to do homework or exercises that I sought”), (b) teachers’ video lecture support (six items, e.g., “The lesson was well explained in the video lecture”), and (c) cooperative learning support (five items, e.g., “When I did group work, I discussed my ideas with other students in my group”). The Cronbach’s alpha values for autonomy support, video lecture support, and cooperative learning support were 0.72, 0.79, and 0.76, respectively, suggesting acceptable internal consistency (Table 2).

Both the learning motivation and instructional behavior questionnaires were self-reported measures, without reverse items included. The original questionnaires were in English and were translated into Khmer using the back-translation technique by two bilingual Cambodian lecturers. After the scales were translated back into English, we compared the Khmer and English versions of the scales to determine whether each item matched the initial meaning. The Khmer version of the scale was subsequently administered to 50 high school students to evaluate each item’s appropriateness and face validity before the data were collected. For all the scales, the students were asked to rate each item on a 5-point Likert scale ranging from 1 (strongly disagree) to 5 (strongly agree). The constructs, dimensions, and items used in this study are shown in Table 1.

### Data analysis

Prior to initiating data analysis, the missing data for each variable included in this study were addressed as follows: (1) responses above 10% missing data were excluded from the study, and (2) variables with missing data below 10%, the missing values were imputed using the observed mean of the corresponding variable. Preliminary analyses and descriptive statistics were used to describe and summarize the data. Skewness and kurtosis values were checked for the normality of the data. Pearson correlation analysis was employed to assess the hypothesized relationships between the variables. Before testing the hypothesized causal relationships, we assessed the construct validity of the measurement model using confirmatory factor analysis (CFA). CFA was performed to validate a measurement model of each aspect of instructional behavior and learning motivation. The convergent validity of the measurement model was verified through average variance extracted (AVE) values. Scale reliability was assessed through composite reliability (CR), Cronbach’s alpha (α), and McDonald’s omega coefficient. The discriminant validity of the constructs was assessed via the heterotrait‒monotrait (HTMT) ratio of correlations, the square root of the AVE, the maximum shared variance (MSV), and the average shared variance (ASV). The accepted level of discriminant validity is an HTMT value less than 0.90^54^, the square root of the AVE for each construct is higher than the correlation coefficient values of the other constructs are^51^, and both the MSV and the ASV are lower than the AVE. The final analysis used structural equation modeling (SEM) with an MLR estimator to test the direct and indirect relationships among the six constructs.

The goodness of fit of the measurement model and the structural equation model was assessed by the following indices and cutoff criteria: chi-square per degrees of freedom (*χ2/df* ≤ 3), comparative fit index (CFI ≥ 0.90), Tucker‒Lewis index (TLI ≥ 0.90), root mean square error of approximation (RMSEA ≤ 0.08), and standardized root mean square residual (SRMR < 0.08)^51,55–58^.

All performances were analyzed with Mplus 8.13 ^59^, the JASP team, and R package software. In the final stage, an independent t-test was employed to compare the mean scores between different groups.

## Results

### Preliminary analysis

Descriptive statistics, including the mean (*M*), standard deviation (*SD*), skewness (*SK*), and kurtosis (*KU*), coupled with α, are displayed in Table 2. This study’s *SK* values were between − 0.523 and 0.263, and the *KU* values were between 0.384 and 1.027, confirming the normality of the data. The mean scores for autonomy support, video lecture support, cooperative learning support, subjective task value, intrinsic motivation, and extrinsic motivation were 3.471 (*SD* = 0.664), 3.434 (*SD* = 0.615), 3.948 (*SD* = 0.465), 4.151 (*SD* = 0.406), 3.934 (*SD* = 0.437), and 3.773 (*SD* = 0.630), respectively (Table 2).

The results of the correlation analysis, as shown in Table 3, indicated that all the correlations were significantly positive. The highest correlation was between subjective task value and intrinsic motivation (*r* = 0.506, *p* < 0.01), whereas the lowest correlation was between video lecture support and subjective task value (*r* = 0.161, *p* < 0.01).

**Table 3:** Pearson correlations (below the diagonal) and the heterotrait–monotrait ratio (above the diagonal) between subaspects.

Constructs		1	2		3		4		5		6	
1	Autonomy support		0.477		0.287		0.303		0.445		0.245	
2	Video lecture support	0.387**			0.327		0.192		0.322		0.249	
3	Cooperative learning support	0.233	0.272**				0.620		0.606		0.271	
4	Subjective task value	0.245**	0.161**		0.469**				0.761		0.423	
5	Intrinsic motivation	0.317**	0.264**		0.427**		0.506**				0.416	
6	Extrinsic motivation	0.205**	0.238**		0.233**		0.324**		0.318**			

** *p* < 0.01

### Confirmatory factor analysis (CFA)

During CFA, a second-order model was established for both the instructional behavior scale and the learning motivation scale. The model fit indices met the required criteria, demonstrating the validity and reliability of the second-order structure for these constructs in the Cambodian context. The acceptable model fit indices are as follows (Table 4): (a) instructional behavior scale, with χ²(77) = 188.377, *χ²/df =* 2.446, CFI = 0.943, TLI = 0.922, SRMR = 0.045, and RMSEA = 0.053 (90% CI: 0.043 to 0.063); and (b) learning motivation scale, with χ²(96) = 163.742, *χ²/df =* 1.706, CFI = 0.964, TLI = 0.955, SRMR = 0.039, and RMSEA = 0.037 (90% CI: 0.027 to 0.047).

**Table 4:** Fit indices for the three models.

Models	χ²	df	*p* value	χ²/df (≤ 3)	CFI (≥ 0.90)	TLI (≥ 0.90)	RMSEA (≤ 0.08).	SRMR (< 0.08)
Measurement model								
Second-order CFA model of the instructional behavior scale	188.377	77	< 0.001	2.446	0.943	0.922	0.053 (90% CI: 0.043 to 0.063)	0.045
Second-order CFA model of the learning motivation scale	163.742	96	< 0.001	1.706	0.964	0.955	0.037 (90% CI: 0.027 to 0.047)	0.039
SEM (effect of each aspect of instructional behaviors on the subscales of learning motivation)								
Baseline Model	4637.291	465	< 0.001	9.973				
Initial Model (Based on the hypothesized framework and including non-significant paths)	951.075	419	< 0.001	2.270	0.872	0.858	0.050 (90% CI: 0.045 to 0.054)	0.054
Model 2 (after removing non-significant paths)	961.111	424	< 0.001	2.267	0.871	0.859	0.050 (90% CI: 0.045 to 0.054)	0.055
Final SEM model presented in this research (modify the model by allowing some of the error values to be related)	822.996	417	< 0.001	1.974	0.903	0.891	0.043 (90% CI: 0.039 to 0.048)	0.051

All standardized factor loading scores were statistically significant and ranged from 0.443 to 0.726 (*p* < 0.01), confirming that the observed variables are reliable indicators of their latent construct^51^.

### Convergent validity and discriminant validity

In this study, the convergent validity of the measurement model was assessed by the AVE and CR. As shown in Table 5, the AVE values were greater than 0.50, which indicates that each construct is accurately measured by its items^60^. Similarly, the CR scores for all the constructs ranged from 0.652 to 0.811, well above the benchmark of 0.70, indicating that the items used to measure the constructs have high reliability^51,56^.

**Table 5:** The CR, AVE, CR, MSV and ASV values of all the constructs.

Constructs	Item	Standardized factor loading	AVE (convergent validity)	CR	$\:\sqrt{\varvec{A}\varvec{V}\varvec{E}}$	MSV	ASV
				(Reliability)	(Discriminant validity)		
Autonomy support	AS1	0.499	0.373	0.701	0.611	0.150	0.081
	AS2	0.550					
	AS3	0.694					
	AS4	0.679					
Video lecture support	VL1	0.541	0.395	0.794	0.629	0.150	0.075
	VL2	0.549					
	VL3	0.759					
	VL4	0.696					
	VL5	0.619					
	VL6	0.579					
Cooperative learning support	SC1	0.724	0.386	0.755	0.621	0.220	0.117
	SC2	0.704					
	SC3	0.600					
	SC4	0.520					
	SC5	0.529					
Subjective task value	SV1	0.443	0.312	0.728	0.558	0.256	0.133
	SV2	0.525					
	SV3	0.602					
	SV4	0.671					
	SV5	0.528					
	SV6	0.553					
Intrinsic motivation	IM1	0.568	0.273	0.652	0.523	0.256	0.142
	IM2	0.553					
	IM3	0.504					
	IM4	0.518					
	IM5	0.464					
Extrinsic motivation	EM1	0.571	0.464	0.811	0.681	0.105	0.072
	EM2	0.709					
	EM3	0.729					
	EM4	0.713					
	EM5	0.671					

Discriminant validity confirms that instruments designed to assess different constructs are independent and do not assess the same fundamental concept^60^. The HTMT value (Table 3) and square root of the AVE for each construct were utilized to assess the discriminant validity of the measurement model. Discriminant validity was established for all the constructs as the HTMT values (ranging from 0.192 to 0.761), and the intercorrelations of the constructs (ranging from 0.161 to 0.506) were less than 0.90^54^ (Table 3). The square root value of the AVE of each construct (ranging from 0.523 to 0.681) was greater than its Pearson correlation coefficient with the other constructs.

### Internal consistency

Cronbach’s alpha and McDonald’s omega coefficients were used to measure the internal consistency of the multidimensional scale. As shown in Table 2, all the factors demonstrated acceptable internal consistency, with both coefficients meeting or exceeding the threshold of 0.60^61^. The reliability results for each factor are as follows: autonomy support (α = 0.72, ω = 0.68), video lecture support (α = 0.79, ω = 0.78), cooperative learning support (α = 0.76, ω = 0.77), subjective task value (α = 0.73, ω = 0.73), intrinsic motivation (α = 0.63, ω = 0.62), and extrinsic motivation (α = 0.81, ω = 0.81).

### Structural equation modeling (SEM)

To investigate the causal relationship of the developed hypothesized structural model between instructional behaviors and learning motivation, this study employed SEM. The analysis focused on the first-order constructs of both instructional behavior and learning motivation scales. However, after adjusting the model according to the suggested modification indices, some paths identified as nonsignificant were not included, with the proposed model being a better-fitting model.

**Fig. 2:**
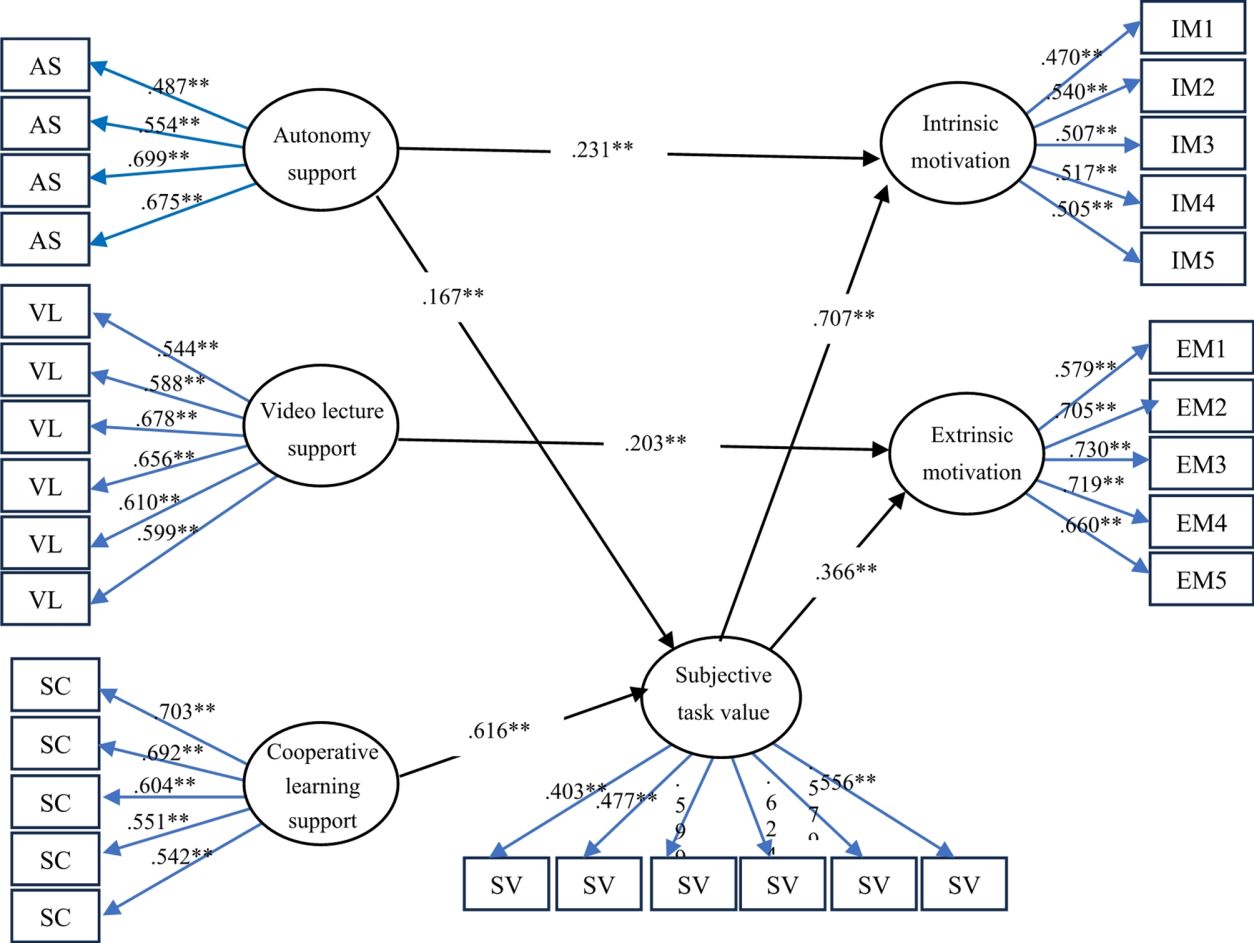
Structural equation modeling (SEM) of the relationships among autonomy support (Autonomy), video lecture support (Video), cooperative learning support (Coop), subjective task value (Task), intrinsic motivation (INMO), and extrinsic motivation (EXMO) (standardized parameter estimates). *Note*: The error terms are omitted for simplicity.

As shown in Tables 4 and 6; Fig. 2, the SEM results revealed an acceptable model fit, with *χ²*(417) = 822.996, *p* < 0.001, *χ²/df* = 1.974, CFI = 0.903, TLI = 0.891, RMSEA = 0.043 (90% CI: 0.039 to 0.048), and SRMR = 0.051. The independent variables explained the proportion of variance in the endogenous latent variables as follows: subjective task value explained approximately 47.4% (*R*^2^ = 0.474), intrinsic motivation explained approximately 67.3% (*R*^*2*^ = 0.673), and extrinsic motivation explained approximately 22.1% (*R*^2^ = 0.221). These findings indicate the robustness of the SEM model, in which the independent variables can efficiently predict the variance in the endogenous latent variables. The standardized path coefficients for direct effects and indirect effects can be summarized as follows:

**Table 6:** Direct and indirect standardized path coefficients.

Hypothesis path	Standardized path coefficient (β)	t value	*p* value
Direct effect			
Autonomy support → Subjective task value	0.167**	2.870	0.004
Cooperative learning support → Subjective task value	0.616**	13.319	< 0.001
Autonomy support → Intrinsic motivation	0.231**	3.698	< 0.001
Subjective task value → Intrinsic motivation	0.707**	13.616	< 0.001
Video lecture support → Extrinsic motivation	0.203**	3.767	< 0.001
Subjective task value → Extrinsic motivation	0.366**	6.877	< 0.001
Indirect effect			
Autonomy support → Subjective task value → Intrinsic motivation	0.118**	2.782	0.005
Cooperative learning support → Subjective task value → Intrinsic motivation	0.436**	9.151	< 0.001
Autonomy support → Subjective task value → Extrinsic motivation	0.061**	2.621	0.009
Cooperative learning support → Subjective task value → Extrinsic motivation	0.226**	6.104	< 0.001

→ = regressed on; ** = p < 0.001.

1. Regarding the direct effect, the SEM results revealed that the construct with the greatest significant positive effect on subjective task value was cooperative learning support (*β* = 0.616, *p* < 0.01), followed by autonomy support (*β* = 0.167, *p* < 0.01), indicating that students who perceive that they receive good cooperative learning support and autonomy support are more likely to have higher subjective task value. The results further demonstrated that subjective task value had the highest standardized positive direct effect on intrinsic motivation (*β* = 0.707, *p* < 0.01), followed by autonomy support (*β* = 0.231, *p* < 0.01), suggesting that students who perceive subjective task value and receive autonomy support tend to have greater intrinsic motivation to learn than others do.

Furthermore, subjective task value (*β* = 0.366, *p* < 0.01) and video lecture support (*β* = 0.203, *p* < 0.01) were positively linked to extrinsic motivation. This finding is surprising because the results showed that students who perceived subjective task value and who had good-quality video lecture support were more likely to have extrinsic motivation, which means action driven by external rewards.

In summary, subjective task value was the only construct that promoted both intrinsic and intrinsic motivations among students in Cambodia.

(2) The indirect effects were analyzed to confirm that subjective task value was a mediator of the relationship between instructional behaviors and motivation types. The findings from the mediation analysis revealed that cooperative learning support had the greatest significant indirect effect on intrinsic motivation (*β* = 0.436, *p* < 0.01) and extrinsic motivation (*β* = 0.226, *p* < 0.01) via subjective task value. This finding means that when students have better collaborative learning when working with groups, they have higher subjective task value, which affects their intrinsic motivation.

Similarly, autonomy support had strong mediating effects on intrinsic motivation (*β* = 0.118, *p* < 0.01) and extrinsic motivation (*β* = 0.061, *p* < 0.01). This finding demonstrates that when students perceive autonomy in learning environments, they tend to develop a greater perception of subjective task value, which leads to an increase in their intrinsic and extrinsic motivations for learning. However, video lecture support did not mediate through subjective task value, suggesting its limited role in fostering intrinsic and extrinsic motivations.

### Results of independent samples t-test

The independent samples t-test results (Table 7) showed significant differences (*p* < 0.01) in perceived instructional behavior, with high school students reporting significantly better perceptions than junior high school students in all three dimensions: autonomy support, video lecture support, and cooperative learning support. When considering the sub-items, the items in which the perceptions of the two groups were not statistically different were AS1, AS2 and SC1 (*p* > 0.01).

**Table 7:** Results of independent samples t-test comparing perceived instructional behaviors across three dimensions between junior high and high school students.

Instructional Behaviors/ Grade Level		*M*		*SD*		Independent-Samples t-Test					
						*t* - value		*p*		Mean Difference	
Autonomy support (AS1 - AS4)	Junior high school	3.396		0.677		-3.090		0.002		-0.182	
	High school	3.578		0.616							
AS1	Junior high school	3.403		0.954		-0.553		0.580		-0.047	
	High school	3.450		0.937							
AS2	Junior high school	3.780		0.877		-1.401		0.162		-0.107	
	High school	3.886		0.808							
AS3	Junior high school	3.180		0.903		-5.528		0.000		-0.394	
	High school	3.573		0.689							
AS4	Junior high school	3.190		1.004		-2.428		0.016		-0.213	
	High school	3.403		0.912							
Video lecture support (VL1 - VL6)	Junior high school	3.237		0.672		-4.293		0.000		-0.249	
	High school	3.486		0.594							
VL1	Junior high school	2.767		1.006		-3.094		0.002		-0.281	
	High school	3.047		0.994							
VL2	Junior high school	3.422		0.994		-3.106		0.002		-0.270	
	High school	3.692		0.933							
VL3	Junior high school	3.007		0.948		-3.008		0.003		-0.254	
	High school	3.261		0.907							
VL4	Junior high school	3.306		0.886		-2.551		0.011		-0.202	
	High school	3.507		0.853							
VL5	Junior high school	3.642		0.810		-4.377		0.000		-0.282	
	High school	3.924		0.628							
VL6	Junior high school	3.813		0.799		-3.081		0.002		-0.196	
	High school	4.009		0.625							
Cooperative learning support (SC1 - SC5)	Junior high school	3.878		0.474		-4.397		0.000		-0.182	
	High school	4.060		0.434							
SC1	Junior high school	3.990		0.664		-1.496		0.135		-0.086	
	High school	4.076		0.597							
SC2	Junior high school	3.844		0.702		-4.848		0.000		-0.269	
	High school	4.114		0.540							
SC3	Junior high school	3.727		0.794		-2.591		0.010		-0.174	
	High school	3.900		0.700							
SC4	Junior high school	3.920		0.616		-4.421		0.000		-0.236	
	High school	4.156		0.551							
SC5	Junior high school	3.907		0.641		-2.610		0.009		-0.146	
	High school	4.052		0.579							

## Discussion

This study investigated how efficient instructional behaviors may be related to student learning motivation using structural equation modeling (SEM) to explore these associations. The SEM findings supported hypothesized relationships by demonstrating that most instructional activities were directly or indirectly associated with endogenous variables.

The study revealed that most instructional behaviors might be associated with all endogenous variables. Subjective task value was directly associated with cooperative learning support. It is more likely that students perceive cooperative learning as an opportunity to engage in meaningful interactions, share ideas during group discussions, and receive teacher-led training in social skills. These experiences ensure that they work positively and accountably with their peers, fostering a sense of responsibility and collaboration. Such an environment may also enhance students connect learning tasks to their interests, set future goals, and recognize the intrinsic value of education in enhancing their competence and achieving academic success. This aligns with research emphasizing the importance of collaborative environments in enhancing task value and student engagement (Johnson & Johnson, 2014; Slavin, 2015). Teachers train students in social skills to ensure that they work positively and accountably during class through positive interactions between peers. This is a reason to attract students to match what they learn, link their interests, set goals for the future, and understand the value that all learning is important for their competence and academic results^62^.

Although cooperative learning support was not directly linked to extrinsic motivation or intrinsic motivation, it appeared to have a significant indirect effect on both dimensions of motivation through subjective task value as a mediating variable. This finding may reflect the hesitation of some students to fully engage during group work, as previously reported by Moon and Ke^37^. The findings suggest that although cooperative learning promotes task valuation, it may not always directly into increased individual motivation, which may be due to different levels of cooperation or cultural and contextual factors that affect group dynamics. However, well-implemented cooperative learning may increase students’ motivation to learn.

Video lecture support was directly associated only with extrinsic motivation, and it was not significantly related to subjective task value or intrinsic motivation, which are important variables for predicting extrinsic motivation. This finding may be explained by students’ perceptions that video lessons provide sufficient support for external needs, such as improving grades or meeting external expectations while alleviating the pressure of comprehension challenges. The results align with those of prior studies indicating that video-based instruction supports performance-oriented outcomes and sustains interest^42,53^. However, the lack of effect on intrinsic motivation or subjective task value indicates that video-based lecture support may lack the interactivity and engagement needed to inspire deeper self-awareness. This reflects the importance of video-based content design that should enhance learner-interactive formats and that content and presentation should engage learners in reflective and meaningful tasks that will motivate them to learn and work in the future^24^.

Autonomy support was found to be the strongest predictor of intrinsic motivation and subjective task value. Encouraging independence makes children feel happier and more valued while learning, which is more valuable for children’s growth than rewards are. These findings are consistent with SDT, which hypothesizes that autonomy enhances intrinsic motivation by fostering a sense of choice and ownership over learning tasks^21^. Teachers who support decision-making and provide opportunities for self-directed learning help learners align their goals with the perceived importance and usefulness of their studies. Such alignment increases intrinsic motivation, leading to curiosity, competence, mastery learning, and sustained academic engagement. These findings corroborate those of previous studies that emphasized the important role of autonomy support in promoting both motivation and academic success^37,38^.

Subjective task value also played an important role in predicting both intrinsic and extrinsic motivations. In this sense, the valuation of self-enjoyment, learning usefulness, and future career goal-related learning might impact how well students’ perceptions increase their curiosity, mastery learning, and self-competence, and it might encourage them to meet externally related requirements set by others that are necessary during academic education, such as grades, rewards, and self-expression^63^. As asserted by previous studies^31^, students might be intrinsically and extrinsically motivated when they value what they learn or do to complete a learning task.

The analysis of indirect effects further revealed that cooperative learning support and autonomy support were significantly associated with intrinsic and extrinsic motivations through the mediating role of subjective task value. These findings highlight the importance of subjective task value in instructional behaviors, aligning with prior studies that identified task value as a critical component for fostering motivation^30,64^. When students perceive cooperative and autonomous support as sufficient, they are more likely to value their learning, connect it with future goals, and develop self-regulatory capabilities. This highlights the need for educators to design instructional strategies that simultaneously promote autonomy, collaboration, and task value to achieve comprehensive motivational outcomes.

However, contrary to expectations, the SEM results in this study found that some expected relationships were nonsignificant, particularly the mediating role of subjective task value in video lecture support and motivation types. Video lecture support did not significantly mediate through subjective task value. This may be due to students’ passive engagement with video-based materials. Research by Brame^65^ and Galatsopoulou et al.^66^ has suggested that video lectures are most effective when students are actively engaged through interactive elements, such as discussions or problem-solving tasks. If students in Cambodia primarily consume video lectures as a one-way delivery of content rather than an interactive experience, this could explain why video lecture support did not strongly influence intrinsic or extrinsic motivation through subjective task value. Additionally, Cambodia’s social and educational environment may play a role in these findings. Studies on Southeast Asian educational contexts (e.g., Sariani et al.^67^ highlight that traditional teacher-centered methods are still dominant, with limited self-regulated and technology-enhanced learning integration. If students are not accustomed to leveraging video lectures for deep learning, their subjective task value for such instructional materials may be lower than expected, resulting in a weak mediation effect. Moreover, external factors such as limited access to high-quality digital resources and variations in technological infrastructure could further explain the weaker role of video lecture support in fostering motivation.

The findings support the notion that instructional behaviors may influence motivation, mainly through enhancing task value^23^, which aligns with SDT’s focus on intrinsic motivation and Pintrich’s view of task value as a key predictor of motivation. This integrated model extends the theoretical understanding of how teacher behaviors can create an environment that fosters intrinsic and extrinsic motivation, suggesting that motivation is not a unidimensional construct but a complex interplay of multiple factors. Additionally, this study contributes to the literature by demonstrating how integrating SDT and Pintrich’s framework can provide a more comprehensive explanation of motivation in educational contexts. The model developed here can be used as a basis for future studies to explore further the interactions between instructional behaviors, student motivation, and academic engagement.

## Future research and limitations

This study focused on secondary and high school students, with a limited sample size that did not represent all regions of Cambodia. Future research should include university students and expand the sample size to increase the generalizability of the findings. Another limitation of this study is its reliance on a cross-sectional approach. Future studies could adopt longitudinal designs to explore the progression of teachers’ instructional practices and students’ motivation over time or conduct multigroup analyses to examine whether the theoretical model holds across different samples.

Moreover, this study did not consider environmental factors such as school policies, school types, or student socioeconomic status, which may influence learning motivation. Future research should incorporate these factors and explore their impact. The use of qualitative methods could also provide deeper insights into the complexities of educational management, teaching practices, and student motivation.

## Conclusion

This study sought efficient instructional behavior to address learning motivation issues after the end of the COVID-19 pandemic in Cambodia. Interestingly, this study reveals efficient instructional behavior, which has three points. First, teachers should provide autonomy support that allows students to make their own decisions through their choices with their learning tasks, group work, and groupmates. Second, the teacher should provide video lecture support linked with students’ interests, be helpful in learning and working on their tasks, provide clear explanations, and make connections with in-class activities. Third, teachers should provide cooperative learning support, such as peer support, peer feedback, and peer teaching, through the responsibility of groups to share their ideas among peers. In summary, further implications might be considered in terms of general education.

## Data Availability

The original data are available on reasonable request from the corresponding author.
